# Prognostic Impact of Circumferential Resection Margin in Minimally Invasive Surgery for Locally Advanced Rectal Cancer: A Multicenter Prospective Study

**DOI:** 10.1002/ags3.70268

**Published:** 2026-08-27

**Authors:** Atsushi Hamabe, Masaaki Ito, Shigeo Toda, Yusuke Suwa, Suguru Hasegawa, Masanori Kotake, Masafumi Inomata, Kazuki Ueda, Atsushi Ogura, Kiichi Sugimoto, Kei Kimura, Koki Otsuka, Tatsuki Noguchi, Koya Hida, Yasumitsu Hirano, Kazushige Kawai, Shigeru Yamagishi, Takeshi Naitoh, Ichiro Takemasa, Japan Society of Laparoscopic Colorectal Surgery

**Affiliations:** ^1^ Department of Gastroenterological Surgery, Graduate School of Medicine The University of Osaka Suita Japan; ^2^ Department of Colorectal Surgery National Cancer Center Hospital East Kashiwa Japan; ^3^ Department of Gastroenterological Surgery Toranomon Hospital Tokyo Japan; ^4^ Department of Surgery, Gastroenterological Center Yokohama City University Medical Center Yokohama Japan; ^5^ Department of Gastroenterological Surgery, Faculty of Medicine Fukuoka University Fukuoka Japan; ^6^ Department of Surgery Koseiren Takaoka Hospital Toyama Japan; ^7^ Department of Gastroenterological and Pediatric Surgery Oita University, Faculty of Medicine Yufu Japan; ^8^ Department of Surgery Kindai University Faculty of Medicine Sakai Japan; ^9^ Division of Surgical Oncology, Department of Surgery Nagoya University Graduate School of Medicine Nagoya Japan; ^10^ Department of Coloproctological Surgery Juntendo University Faculty of Medicine Tokyo Japan; ^11^ Division of Lower Gastrointestinal Surgery, Department of Gastroenterological Surgery Hyogo Medical University Nishinomiya Japan; ^12^ Department of Surgery Fujita Health University, School of Medicine Toyoake Japan; ^13^ Department of Gastroenterological Surgery Cancer Institute Hospital of the Japanese Foundation for Cancer Research Tokyo Japan; ^14^ Department of Surgery, Graduate School of Medicine Kyoto University Kyoto Japan; ^15^ Department of Gastroenterological Surgery Saitama Medical University International Medical Center Hidaka Japan; ^16^ Department of Surgery Tokyo Metropolitan Cancer and Infectious Diseases Center Komagome Hospital Tokyo Japan; ^17^ Department of Surgery Fujisawa Municipal Hospital Fujisawa Kanagawa Japan; ^18^ Department of Lower Gastrointestinal Surgery Kitasato University School of Medicine Sagamihara Japan; ^19^ Department of Gastroenterological Surgery Osaka International Medical and Science Center, Osaka Keisatsu Hospital Osaka Japan

**Keywords:** circumferential resection margin, laparoscopic surgery, long‐term outcome, rectal cancer, robotic surgery

## Abstract

**Aim:**

Circumferential resection margin (CRM) status is a well‐established prognostic factor in rectal cancer surgery; however, its long‐term oncological relevance has not been prospectively validated in Japan, where upfront surgery has long been widely used. This study assessed the prognostic impact of CRM in patients with locally advanced rectal cancer treated with minimally invasive surgery.

**Methods:**

The PRODUCT trial was a multicenter, prospective, observational study conducted at 18 institutions in Japan. Patients aged ≥ 20 years with clinical stage II or III rectal adenocarcinoma located within 12 cm from the anal verge and scheduled for laparoscopic or robotic surgery were enrolled. CRM positivity was defined as a margin of ≤ 1 mm. Long‐term outcomes were analyzed using the Kaplan–Meier method and Cox proportional hazards regression models.

**Results:**

Among 303 eligible patients, CRM positivity was observed in 26 (8.6%). The 3‐year recurrence‐free survival (RFS) rate was significantly lower in the CRM‐positive group than in the CRM‐negative group (61.5% vs. 82.3%; log‐rank *p* = 0.003). CRM positivity was independently associated with worse RFS (hazard ratio, 2.35; 95% confidence interval, 1.17–4.71; *p* = 0.016). In patients with pT3–T4 tumors, recurrence rates were significantly higher in CRM‐positive cases than in CRM‐negative cases (47.6% vs. 21.2%; *p* = 0.013). CRM status was not significantly associated with overall survival or cumulative incidence of local recurrence (*p* = 0.057 and *p* = 0.706, respectively).

**Conclusions:**

CRM positivity is an adverse prognostic factor in Japanese patients with locally advanced rectal cancer undergoing MIS, underscoring the clinical importance of standardized CRM assessment.

**Trial Registration:**

Clinical Trials Registry, Identification Number: UMIN00034365

## Graphical abstract

This multicenter prospective study provides the first Japanese evidence that circumferential resection margin positivity after minimally invasive surgery is associated with poorer long‐term outcomes in locally advanced rectal cancer. As the cohort predominantly included patients treated with upfront surgery, these findings provide important evidence to guide individualized treatment strategies.

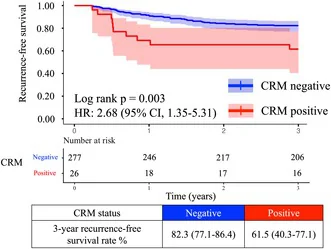

## 1 Introduction

Rectal cancer has long been characterized by substantial risks of both local recurrence and disease‐related mortality [1]. To enhance local disease control, preoperative chemoradiotherapy (CRT) became widely adopted as a standard approach. More recently, treatment strategies have shifted toward total neoadjuvant therapy (TNT), which integrates systemic chemotherapy into the preoperative setting to improve control of distant metastases, increase rates of clinical complete response (cCR), and potentially broaden eligibility for nonoperative management (NOM) [2, 3, 4].

Despite these advances in multimodal therapy, surgical resection remains fundamental to curative treatment for rectal cancer. Among pathological quality indicators, assessment of the circumferential resection margin (CRM) in resected specimens is internationally regarded as a key determinant of oncological adequacy and long‐term prognosis [5]. In Japan, routine CRM evaluation has historically been challenging because of conventional pathological processing practices, including extensive mesorectal trimming for lymph node retrieval and routine opening of the bowel to assess tumor morphology [6]. To overcome these limitations, we previously established a standardized and reproducible method for CRM measurement applicable to routine clinical practice in Japan [6]. Using this methodology, we conducted the PRODUCT trial, a multicenter prospective study enrolling 300 patients with advanced rectal cancer treated with minimally invasive surgery (MIS), and reported short‐term surgical outcomes based on CRM status [7]. That study demonstrated a CRM positivity rate of 8.6%, enabling, for the first time, benchmarking of Japanese rectal cancer surgery against international standards.

The present study extends the PRODUCT trial by focusing on long‐term oncological outcomes. Specifically, we aimed to evaluate long‐term outcomes in this cohort and to clarify the association between pathological CRM status and oncological prognosis.

## 2 Methods

### 2.1 Study Design

This multicenter prospective observational study was conducted across 18 institutions affiliated with the Japan Society of Laparoscopic Colorectal Surgery. Patients aged 20 years or older with histologically confirmed rectal adenocarcinoma located within 12 cm from the anal verge and classified as clinical Stages II or III (T3N0M0 or T1–4aN1–2 M0) were eligible. According to Japanese classification criteria, lateral lymph nodes were considered regional, and isolated lateral lymph node metastasis was categorized as Stage III disease. Tumors assessed as T4a were included provided that invasion of adjacent organs was absent and extension below the peritoneal reflection was limited.

Clinical staging was performed using rectal magnetic resonance imaging (MRI) and was determined prior to neoadjuvant therapy when administered. Decisions regarding neoadjuvant treatment, surgical approach, and lateral lymph node dissection (LLND) were performed according to institutional practice. Patients with active double malignancies (synchronous cancer or metachronous cancer with a disease‐free interval < 5 years), direct invasion into adjacent organs, or psychiatric disorders that could compromise protocol adherence were excluded. The planned sample size was 300 patients. All procedures were performed by surgeons certified under the Endoscopic Surgical Skill Qualification System of the Japan Society for Endoscopic Surgery. Both conventional laparoscopic and robotic‐assisted approaches were permitted.

The study protocol was approved by the institutional review boards of all participating centers. Written informed consent was obtained from all patients. The study was registered in the UMIN Clinical Trials Registry (UMIN00034365).

### 2.2 Outcomes

The primary endpoint was pathological CRM status, as reported in the initial analysis [7]. CRM negativity was defined as a distance greater than 1 mm between the deepest tumor invasion and the resection margin. Secondary endpoints included total mesorectal excision (TME) quality, perioperative outcomes, pathological findings, recurrence‐free survival (RFS), overall survival (OS), and the cumulative incidence of local recurrence. In the present analysis, local recurrence was defined as the first documented recurrence within the pelvis. Postoperative surveillance consisted of serum carcinoembryonic antigen (CEA) and carbohydrate antigen 19–9 (CA19‐9) measurements every 3 months for the first 3 years and every 6 months for the subsequent 2 years. Computed tomography was performed every 6 months for 5 years. Colonoscopy was performed at 1 and 3 years after surgery.

### 2.3 Statistical Analysis

All statistical analyses were conducted using the R software (version 4.5.1). Categorical variables are summarized as frequencies, and continuous variables as medians with interquartile ranges. Group comparisons were performed using Fisher's exact test. Time‐to‐event outcomes were analyzed using the Kaplan–Meier (KM) method, with group differences assessed by the log‐rank test. Hazard ratios with corresponding 95% confidence intervals were calculated using Cox proportional hazards regression models in both univariable and multivariable settings. Variables for the multivariable Cox proportional hazards model were selected based on the results of univariable analyses and clinical relevance. To reduce the risk of overfitting, the number of covariates was restricted in consideration of the number of RFS events. The cumulative incidence of local recurrence was estimated using a competing‐risk approach. Because only the site of first recurrence was collected in this study, first recurrence at non‐local sites was treated as a competing event. Death without local recurrence was also treated as a competing event. The cumulative incidence function was compared between groups using Gray's test. The association between CRM status and local recurrence was evaluated using a Fine–Gray subdistribution hazards model, and subdistribution hazard ratios (sHRs) with 95% confidence intervals (CIs) were calculated.

## 3 Results

### 3.1 Patient Background and Tumor Characteristics

**FIGURE 1:**
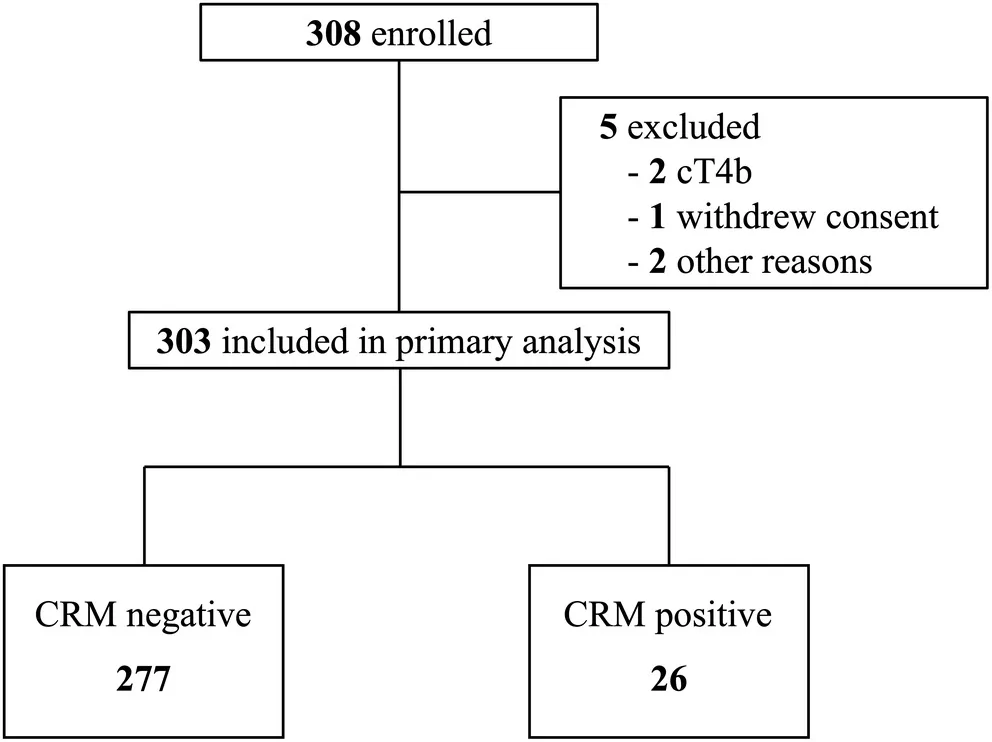
Patient flow diagram of the PRODUCT trial. CRM, circumferential resection margin.

Between August 2018 and January 2020, 308 patients were enrolled (Figure 1). Five patients were excluded after enrolment: two due to adjacent organ invasion identified at baseline diagnosis, one due to withdrawal of consent, and two for other reasons. Consequently, 303 patients were included in the primary analysis.

Patient and tumor characteristics are summarized in Table 1. At baseline, 139 patients had Stage II disease and 164 had Stage III disease, with suspected lateral lymph node metastasis in 30 cases. The median distance from the lower tumor margin to the anal verge was 70 mm (IQR, 45–100 mm). Regarding neoadjuvant treatment, 213 patients underwent upfront surgery, while 90 patients received neoadjuvant therapy, including CRT (*n* = 27), neoadjuvant chemotherapy (NAC; *n* = 45), and TNT (*n* = 18).

**TABLE 1:** Patient characteristics and clinical features.

Patient characteristics (*N* = 303)	
Age, years	66 (56–74)
Male sex	202 (66.7)
BMI, kg/m^2^	22.0 (20.2–24.1)
ECOG‐PS	
0–1	288 (95.0)
2	15 (5.0)
Tumor characteristics at initial examination (*N* = 303)	
Distance from the anal verge, mm	70 (45–100)
Location of the center of the tumor	
Anterior	85 (28.1)
Posterior	74 (24.4)
Right	31 (10.2)
Left	64 (21.1)
Circular	49 (16.1)
Tumor stage at baseline	
cT1	1 (0.3)
cT2	11 (3.6)
cT3	262 (86.5)
cT4a	29 (9.6)
Nodal status at baseline	
cN0	139 (45.9)
cN1	129 (42.6)
cN2	35 (11.6)
Suspected metastasis to lateral lymph node	30 (9.9)
Clinical stage at baseline	
Stage II	139 (45.9)
Stage III	164 (54.1)
Neoadjuvant treatmenttherapy	
None	213 (70.3)
(C)RT	27 (8.9)
NAC	45 (14.9)
TNT	18 (5.9)
Tumor characteristics after neoadjuvant therapy (*N* = 90)	
Tumor stage after neoadjuvant therapy	
ycT0 or cCR	1 (1.1)
ycT1	3 (3.3)
ycT2	18 (20.0)
ycT3	60 (66.7)
ycT4a	5 (5.6)
Not assessed	3 (3.3)
Nodal status after neoadjuvant therapy	
ycN0	58 (64.4)
ycN1	23 (25.6)
ycN2	6 (6.7)
Not assessed	3 (3.3)
Suspected metastasis to lateral lymph node	17 (18.9)
cCR	1 (1.1)

*Note:* Values are given as median (IQR) or *n* (%).

Abbreviations: BMI, body mass index; cCR, clinical complete response; CRT, chemoradiotherapy; ECOG, Eastern Cooperative Oncology Group; IQR, interquartile range; NAC, neoadjuvant chemotherapy; PS, Performance Status; RT, radiotherapy; TNT, total neoadjuvant therapy.

### 3.2 Surgical and Pathological Outcomes

Surgical outcomes are summarized in Table 2. Conventional laparoscopic surgery was performed in 167 patients, and robotic surgery in 136 patients. The operative procedures included anterior resection (*n* = 240), intersphincteric resection (*n* = 40), abdominoperineal resection (*n* = 19), and Hartmann's procedure (*n* = 4). A diverting stoma was constructed in 177 cases.

**TABLE 2:** Surgical outcomes.

Approach	
CLS	167 (55.1)
RALS	136 (44.9)
TaTME performed	78 (25.7)
Procedure	
LAR	240 (79.2)
ISR	40 (13.2)
APR	19 (6.3)
Hartmann	4 (1.3)
LLND	
None	213 (70.3)
Bilateral	76 (25.1)
Unilateral	14 (4.6)
Diverting stoma created	177 (62.1)
Operative duration, min	336 (256–470)
Operative duration excluding LLND cases, min	320 (256–393)
Blood loss, ml	12 (5–50)
Blood loss excluding LLND cases, ml	7 (1–50)
Open conversion	1 (0.3)
30‐day mortality	0 (0.0)
Length of hospital stay, days	14 (12–21)
Grade 3–4 postoperative complications	
Leakage	22 (7.3)
Bowel obstruction	8 (2.6)
Ileus	10 (3.3)
SSI (excluding leak)	10 (3.3)
Urological complication	10 (3.3)
Cardiovascular complication	4 (1.3)
Respiratory complication	3 (1.0)
Hemorrhage	3 (1.0)
Others	10 (3.3)

*Note:* Values are given as median (IQR) or *n* (%).

Abbreviations: APR, abdominoperineal resection; CLS, conventional laparoscopic surgery; IQR, interquartile range; ISR, intersphincteric resection; LAR, low anterior resection; LLND, lateral lymph node dissection; RALS, robotic‐assisted laparoscopic surgery; SSI, surgical site infection; TaTME, transanal total mesorectal excision.

Pathological findings are presented in Table 3. The median pathological CRM, the primary endpoint of the study, was 4.0 mm (IQR, 2.1–8.0 mm). CRM was ≤ 1 mm in 26 patients, yielding a positivity rate of 8.6%. Pathological complete response (pCR) was confirmed in 14 patients, corresponding to a pCR rate of 15.6%. Pathological staging revealed 2, 76, 104, and 107 patients with Stages 0, I, II, and III disease, respectively. Lateral lymph node metastasis was pathologically confirmed in 12 patients, including 3 with isolated lateral lymph node involvement. A positive distal resection margin was observed in one patient.

**TABLE 3:** Pathological findings.

Outcomes	All cases (*N* = 303)	Neoadjuvant therapy	
		No (*N* = 213)	Yes (*N* = 90)
CRM, mm	4.0 (2.1–8.0)	4.0 (2.2–8.0)	4.2 (2.0–7.3)
With negative margin (> 1 mm)	277 (91.4)	197 (92.5)	80 (88.9)
With positive margin (≤ 1 mm)	26 (8.6)	16 (7.5)	10 (11.1)
Tumoral region with CRM‐positivity (*N* = 26)			
Main tumor	18 (69.2)	9 (56.3)	9 (90.0)
Lymph node	6 (23.1)	5 (31.3)	1 (10.0)
Tumor nodule without residual lymph node structure	1 (3.8)	1 (6.3)	0 (0)
Intra‐lymphatic duct invasion	1 (3.8)	1 (6.3)	0 (0)
Total mesorectal excision			
Complete	293 (96.7)	205 (96.2)	88 (97.8)
Nearly complete	10 (3.3)	8 (3.8)	2 (2.2)
Incomplete	0 (0.0)	0 (0)	0 (0)
Tumor size, mm	36 (30–45)	35 (30–45)	38 (30–45)
Pathological tumor stage			
pT0	16 (5.3)	0 (0)	16 (17.8)
pTis	2 (0.7)	0 (0)	2 (2.2)
pT1	10 (3.3)	6 (2.8)	4 (4.4)
pT2	84 (28.1)	59 (27.7)	25 (27.8)
pT3	176 (58.9)	139 (65.3)	37 (41.1)
pT4a	14 (4.7)	9 (4.2)	5 (5.6)
pT4b	1 (0.3)	0 (0)	1 (1.1)
Pathological nodal stage			
pN0	196 (64.7)	134 (62.9)	62 (68.9)
pN1	74 (24.4)	51 (23.9)	23 (25.6)
pN2	33 (10.9)	28 (13.1)	5 (5.6)
Metastasis to lateral lymph node	12 (4.0)	4 (1.9)	8 (8.9)
Pathological stage			
pCR	14 (4.6)	—	14 (15.6)
Stage 0	2 (0.7)	0 (0)	2 (2.2)
Stage I	76 (25.1)	50 (23.5)	26 (28.9)
Stage II	104 (34.3)	84 (39.4)	20 (22.2)
Stage III	107 (35.3)	79 (37.1)	28 (31.1)
PRM, mm	120 (100–150)	120 (100–150)	130 (105–163)
DRM, mm	20 (15–30)	25 (15–31)	20 (12–30)

*Note:* Values are given as median (IQR) or *n* (%).

Abbreviations: CR, complete response; CRM, circumferential resection margin; DRM, distal resection margin; IQR, interquartile range; PRM, proximal resection margin.

### 3.3 Characteristics and Recurrence Patterns According to CRM Status

Baseline, surgical, pathological characteristics, and recurrence patterns according to CRM status are shown in Table 4. There were no statistically significant differences in age, sex, BMI, clinical T category, clinical nodal status, tumor location, or use of neoadjuvant therapy between the CRM‐negative and CRM‐positive groups. However, CRM‐positive patients tended to have pathologically more advanced T or N stages (*p* = 0.0568 and 0.0522, respectively), and have a higher rate of lateral lymph node dissection than CRM‐negative patients (46.2% vs. 28.2%; *p* = 0.0714). Among patients who underwent lateral lymph node dissection, bilateral dissection was performed in all CRM‐positive patients, compared with 64 of 78 CRM‐negative patients. Pathologically confirmed lateral lymph node metastasis was more frequent in the CRM‐positive group than in the CRM‐negative group (15.4% vs. 2.9%; *p* = 0.0132).

**TABLE 4:** Baseline, surgical, pathological characteristics, and recurrence patterns according to CRM status.

	CRM‐negative (*N* = 277)	CRM‐positive (*N* = 26)	*p*
Patient characteristics			
Median age (IQR), years	66 (56–74)	67 (53–76)	0.7895
Sex, *n* (%)			0.1907
Male	188 (67.9)	14 (53.8)	
Female	89 (32.1)	12 (46.2)	
Median BMI (IQR), kg/m^2^	22.0 (20.4–24.1)	20.7 (19.5–23.7)	0.1906
Tumor characteristics at initial examination			
Tumor stage, *n* (%)			0.0876 (cT1‐3 vs. cT4a)
cT1	1 (0.4)	0 (0.0)	
cT2	11 (4.0)	0 (0.0)	
cT3	241 (87.0)	21 (80.8)	
cT4a	24 (8.7)	5 (19.2)	
Nodal status, *n* (%)			0.8375 (cN‐ vs. cN+)
cN0	128 (46.2)	11 (42.3)	
cN1	117 (42.2)	12 (46.2)	
cN2	32 (11.6)	3 (11.5)	
The lower border of the tumor			0.8381
Above the peritoneal reflection	139 (50.2)	12 (46.2)	
Below the peritoneal reflection	138 (49.8)	14 (53.8)	
Neoadjuvant therapy			0.3691
Yes	80 (28.9)	10 (38.5)	
No	197 (71.1)	16 (61.5)	
Surgery‐related results			
Approach			
Laparoscopy	154 (55.6)	13 (50.0)	0.6812
Robot	123 (44.4)	13 (50.0)	
Procedure			0.5995
LAR	221 (79.8)	19 (73.1)	
ISR	36 (13.0)	4 (15.4)	
APR	16 (5.8)	3 (11.5)	
Hartmann	4 (1.4)	0 (0.0)	
Lateral lymph node dissection	78 (28.2)	12 (46.2)	0.0714
Unilateral	14	0	
Bilateral	64	12	
TaTME			
Yes	74 (26.7)	4 (15.4)	0.2479
No	203 (73.3)	22 (84.6)	
Pathological results			
CRM (IQR), mm	5.0 (3.0–8.0)	0.78 (0.44–1.0)	< 0.0001
Tumor size (IQR), mm	35 (30–45)	40 (30–45)	0.2270
Pathological tumor stage			0.0568 (≤ pT2 vs. ≤ pT3)
pT0	16 (5.8)	0 (0.0)	
pTis	2 (0.7)	0 (0.0)	
pT1	10 (3.6)	0 (0.0)	
pT2	79 (28.5)	5 (19.2)	
pT3	159 (57.4)	17 (65.4)	
pT4a	11 (4.0)	3 (11.5)	
pT4b	0 (0.0)	1 (3.8)	
Pathological nodal stage			0.0522 (pN+ vs. pN‐)
pN0	184 (66.4)	12 (46.2)	
pN1	66 (23.8)	8 (30.8)	
pN2	27 (9.7)	6 (23.1)	
Metastasis to lateral lymph node	8 (2.9)	4 (15.4)	0.0132
PRM (IQR), mm	120 (100–150)	120 (100–150)	0.9077
DRM (IQR), mm	24 (15–32)	20 (15–22)	0.0730
Sites of first recurrence (multiple sites allowed)			
Local	7 (2.5)	1 (3.9)	0.5164
Liver	11 (4.0)	3 (11.5)	0.1081
Lung	27 (9.8)	4 (15.4)	0.3210
Peritoneum	0 (0.0)	2 (7.7)	0.0071
Extrapelvic lymph nodes	5 (1.8)	0 (0.0)	1.0000
Others	4 (1.4)	2 (7.7)	0.0858

*Note:* Values are given as median (IQR) or *n* (%). MRI was not performed in 13 cases.

Abbreviations: APR, abdominoperineal resection; BMI, body mass index; CRM, circumferential resection margin; IQR, interquartile range; ISR, intersphincteric resection; LAR, low anterior resection; TaTME, transanal total mesorectal excision.

Regarding recurrence patterns, local recurrence occurred in 7 patients in the CRM‐negative group and 1 patient in the CRM‐positive group. The frequencies of liver, lung, peritoneal, and other distant recurrences were numerically higher in the CRM‐positive group, although statistical significance was not consistently observed because of the small number of events.

### 3.4 Long‐Term Outcomes

**FIGURE 2:**
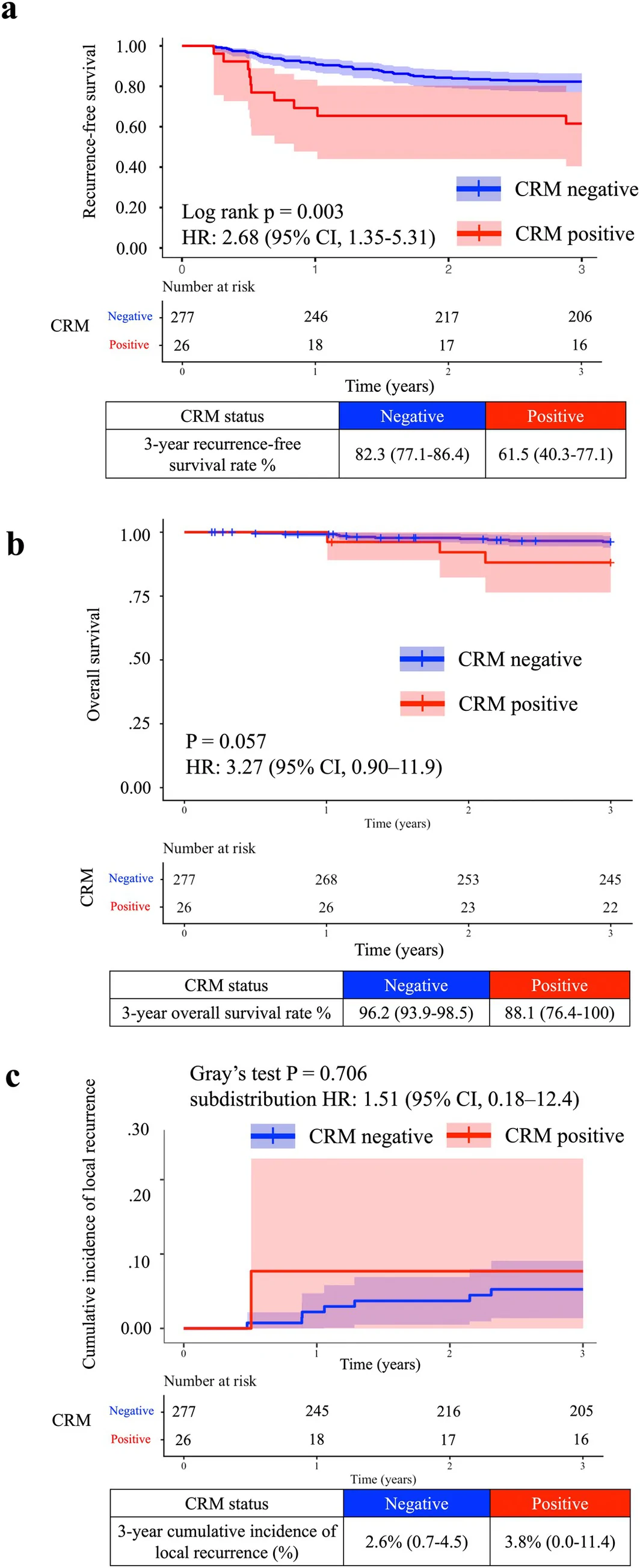
Long‐term outcomes stratified by CRM status. (a) The 3‐year recurrence‐free survival stratified by CRM status. (b) The 3‐year overall survival stratified by CRM status. (c) The cumulative incidence of local recurrence stratified by CRM status. CRM, circumferential resection margin.

The median follow‐up time was 1603 (1476–1709) days. The 3‐year recurrence‐free survival (RFS) rates were 82.3% in the CRM‐negative group and 61.5% in the CRM‐positive group, with significantly better outcomes observed in CRM‐negative patients (log‐rank *p* = 0.003) (Figure 2a). CRM positivity was associated with a hazard ratio (HR) of 2.68 (95% CI, 1.35–5.31; *p* = 0.003). Univariable Cox proportional hazards analysis identified CRM positivity (*p* = 0.005), pT3 or deeper invasion (*p* = 0.001), and pathological lymph node positivity (*p* < 0.001) as significant predictors of shorter RFS, while age ≥ 65 years and robotic surgery showed a nonsignificant trend toward worse RFS (*p* = 0.077 and 0.079, respectively) (Table 5). Other factors, including sex, ECOG performance status, neoadjuvant treatment, and tumor location were not significantly associated with RFS. In multivariable Cox analysis including five clinically relevant variables (CRM status, age category, pT classification, nodal status, and surgical approach), CRM positivity (HR 2.35; 95% CI, 1.17–4.71; *p* = 0.016), pT3 or deeper invasion (HR 2.43; 95% CI, 1.20–4.90; *p* = 0.013), pathological lymph node positivity (HR 2.59; 95% CI, 1.49–4.49; *p* < 0.001) and robotic approach (HR 1.74; 95% CI, 1.03–2.94; *p* = 0.039) were identified as independent adverse prognostic factors for RFS. Given that approximately 35% of enrolled patients had pathological T2 or less disease, a subgroup analysis limited to pT3 and pT4 tumors was conducted to evaluate the prognostic impact of CRM in deeply invasive cancers (Table 6). Recurrence rates were significantly higher in CRM‐positive tumors (47.6%) than in CRM‐negative tumors (21.2%) (*p* = 0.013).

**TABLE 5:** Univariable and multivariable Cox proportional hazards analyses for recurrence‐free survival.

	Univariable analysis		Multivariable analysis	
	HR (95% CI)	*p*	HR (95% CI)	*p*
Sex Male (vs Female)	1.53 (0.85–2.76)	0.157		
Age ≥ 65 years (vs 65 < years)	1.61 (0.95–2.73)	0.077	1.68 (0.979–2.87)	0.060
ECOG PS 1–2 (vs PS 0)	0.96 (0.41–2.24)	0.925		
Lower border of the tumor below peritoneal reflection (vs above peritoneal reflection)	1.52 (0.89–2.57)	0.124		
Neoadjuvant therapy yes (vs no)	1.27 (0.74–2.19)	0.388		
Robot (vs Laparoscopy)	1.59 (0.94–2.70)	0.079	1.74 (1.03–2.94)	**0.039**
pT3‐4 (vs pT0‐2)	3.09 (1.56–6.12)	**0.001**	2.43 (1.20–4.90)	**0.013**
pN positive (vs negative)	3.32 (1.95–5.66)	**< 0.001**	2.59 (1.49–4.49)	**< 0.001**
pCRM positive (vs negative)	2.68 (1.35–5.31)	**0.005**	2.35 (1.17–4.71)	**0.016**

*Note:* Bold values indicate statistical significance with a *p* value of < 0.05.

**TABLE 6:** Subgroup analysis limited to pT3–T4 tumors.

	pCRM negative (*N* = 170)	pCRM positive (*N* = 21)	*p*
Recurrence rate	21.2%	47.6%	0.013

Abbreviation: CRM, circumferential resection margin.

The 3‐year overall survival rates were 96.2% in the CRM‐negative group and 88.1% in the CRM‐positive group, without a statistically significant difference (*p* = 0.057). The HR for CRM positivity was 3.27 (95% CI, 0.90–11.9; *p* = 0.072) (Figure 2b). The 3‐year cumulative incidence of local recurrence did not differ significantly between the CRM‐negative and CRM‐positive groups (2.6% [95% CI, 0.7–4.5] vs. 3.8% [95% CI, 0.0–11.4]; Gray's test *p* = 0.706). In the Fine–Gray model, CRM positivity was not significantly associated with local recurrence (sHR, 1.51; 95% CI, 0.18–12.4; *p* = 0.704) (Figure 2c).

## 4 Discussion

In this multicenter prospective study, we evaluated the oncological outcomes of minimally invasive surgery for locally advanced rectal cancer in Japan using pathological CRM assessment. Our findings demonstrate that CRM positivity is a significant adverse prognostic factor, particularly associated with distant recurrence and poorer long‐term outcomes. To our knowledge, these results provide the first prospective report evaluating the long‐term prognostic significance of CRM in the Japanese cohort.

As rectal cancer treatment strategies have evolved, including the increasing adoption of TNT, surgical procedures have become more technically demanding [8]. Accordingly, objective assessment of surgical quality based on internationally accepted criteria has become increasingly important. CRM status represents one of the most established pathological indicators for evaluating surgical adequacy and long‐term prognosis. By confirming the prognostic significance of CRM in a prospective Japanese cohort, the present study supports the incorporation of CRM assessment into the evaluation of preoperative treatment strategies in Japan. The PRODUCT trial previously standardized CRM measurement and reported a CRM positivity rate of 8.6% in a multicenter MIS cohort [7]. The current long‐term analysis further validates the clinical relevance of this measurement methodology by demonstrating a clear association between CRM positivity and adverse oncological outcomes. Importantly, more than 70% of patients in this cohort underwent upfront surgery, distinguishing this population from Western cohorts in which neoadjuvant treatment is more commonly applied.

Historically, the prognostic importance of CRM was first recognized before the widespread adoption of TME [9] and was subsequently confirmed in the Dutch TME trial [10]. Since then, advances in preoperative treatment strategies, including routine use of CRT and expansion of TNT, have been achieved [11, 12]. Even in this evolving treatment landscape, CRM has consistently remained a significant prognostic factor, including in patients treated with TNT [8]. In parallel, laparoscopic surgery has been increasingly adopted for locally advanced rectal cancer, with randomized trials such as COLOR II and COREAN demonstrating oncological equivalence to open surgery [13, 14]. Although initial concerns regarding specimen quality were raised in the ALaCaRT and ACOSOG Z6051 trials [15, 16], subsequent long‐term follow‐up confirmed that laparoscopic surgery was not associated with inferior oncological outcomes, while CRM continued to serve as a strong predictor of prognosis [17, 18]. Because TME was established before the widespread implementation of neoadjuvant therapy, the present study represents one of the first large‐scale prospective evaluations of CRM as a prognostic factor in MIS‐treated locally advanced rectal cancer with a substantial proportion of upfront surgery cases. As treatment strategies become increasingly individualized, balancing intensified preoperative therapy against treatment‐related toxicity has become a major clinical challenge [19, 20]. Although TNT aimed at NOM has shown promising results, recent studies have also demonstrated the prognostic utility of circulating tumor DNA in upfront surgery cohorts [21]. In this context, establishing the prognostic significance of CRM in MIS‐treated upfront surgery cases is clinically meaningful.

Consistent with long‐term results from the ALaCaRT trial, pathological T and N factors were also identified as prognostic indicators for RFS in the present study [17]. In addition, CRM positivity was associated with significantly higher recurrence rates in pT3–T4 tumors, emphasizing the importance of appropriate dissection planes to secure CRM during surgery for deeply invasive disease. Achieving an adequate CRM may require en bloc resection of adjacent structures, which must be carefully balanced against postoperative functional outcomes. Accurate preoperative tumor assessment is therefore essential. Although MRI is regarded as mandatory for preoperative staging of rectal cancer in Western guidelines, it is not routinely recommended in Japanese guidelines [2, 3, 22]. The present findings may reinforce the importance of accurate preoperative imaging assessment, including MRI.

Although CRM involvement is recognized as an important risk factor for local recurrence after rectal cancer surgery, CRM positivity was significantly associated with worse RFS but not with the cumulative incidence of local recurrence in the present study [5, 23]. This discrepancy should be interpreted cautiously. In the additional CRM‐stratified analysis, CRM‐positive patients tended to undergo lateral lymph node dissection more frequently, and all CRM‐positive patients who underwent lateral lymph node dissection received bilateral dissection. Pathologically confirmed lateral lymph node metastasis was also more frequent in the CRM‐positive group. These findings suggest that CRM positivity may reflect more advanced pelvic disease rather than simply inadequate radial resection margins. The low incidence of local recurrence despite CRM positivity may partly relate to the Japanese treatment strategy of performing lateral lymph node dissection in selected patients with suspected lateral lymph node disease [22]. However, this interpretation remains speculative, and the limited number of local recurrence events, with only one event in the CRM‐positive group and seven events in the CRM‐negative group, restricts statistical power. These findings suggest that the adverse association between CRM positivity and RFS in this cohort was more clearly reflected in systemic recurrence than in local recurrence. Nevertheless, because the number of local recurrence events was limited, the present study was not sufficiently powered to draw firm conclusions regarding the relationship between CRM positivity and local failure.

The adverse association between robotic approach and worse RFS in this cohort should be interpreted cautiously, because the PRODUCT trial was conducted during an early phase of robotic adoption in Japan, when many participating surgeons were already well experienced in laparoscopic surgery but may still have had limited experience with robotic rectal surgery. Based on this background, we hypothesized that robotic surgery, with its articulated instruments and enhanced precision, might reduce CRM positivity under strict surgeon credentialing [24]. This hypothesis led to the VITRUVIANO trial, which demonstrated a lower CRM positivity rate of 4.6%. The ROLARR trial did not demonstrate a clear advantage of robotic surgery in terms of achieving an adequate CRM [25]. However, this may be partly attributable to the wide variation in surgeons' technical experience in that study. In contrast, the VITRUVIANO trial restricted operating surgeons to those with experience of more than 40 procedures, which may have contributed to the observed impact on CRM outcomes. Similarly, the REAL trial conducted in China reported lower CRM positivity rates and improved oncological outcomes in patients undergoing robotic surgery compared with laparoscopic surgery [26, 27]. Long‐term outcome data from the VITRUVIANO trial are awaited to further clarify the prognostic implications of CRM.

Several limitations of this study should be acknowledged. First, both laparoscopic and robotic approaches were included, introducing heterogeneity in surgical technique. Secondly, treatment strategies, including neoadjuvant therapy and lateral lymph node dissection, were based on institutional practice rather than a uniform protocol. Thirdly, although the number of covariates in the multivariable model was restricted in consideration of the number of RFS events, the limited number of CRM‐positive patients may have affected the precision of the estimated association between CRM status and RFS. Finally, because the cohort consisted predominantly of patients undergoing upfront surgery, the generalizability of these findings to treatment settings with more routine use of neoadjuvant therapy or total neoadjuvant therapy may be limited.

In conclusion, this multicenter prospective study demonstrates that CRM status is a critical prognostic factor associated with long‐term outcomes in patients with locally advanced rectal cancer treated with minimally invasive surgery in Japan. Given the substantial proportion of upfront surgery cases included, these findings provide clinically relevant evidence to support individualized treatment strategies in contemporary rectal cancer management.

## Author Contributions

**Atsushi Hamabe:** conceptualization, writing – original draft, methodology, data curation, investigation, validation, formal analysis, visualization. **Shigeo Toda:** investigation, writing – review and editing, data curation. **Kiichi Sugimoto:** data curation, investigation, writing – review and editing. **Koya Hida:** data curation, investigation, writing – review and editing. **Masanori Kotake:** investigation, writing – review and editing, data curation. **Masaaki Ito:** conceptualization, investigation, writing – review and editing. **Kei Kimura:** data curation, investigation, writing – review and editing. **Suguru Hasegawa:** investigation, writing – review and editing, data curation. **Koki Otsuka:** data curation, investigation, writing – review and editing. **Yasumitsu Hirano:** data curation, investigation, writing – review and editing. **Masafumi Inomata:** investigation, writing – review and editing, data curation. **Atsushi Ogura:** data curation, investigation, writing – review and editing. **Ichiro Takemasa:** conceptualization, data curation, writing – review and editing, supervision, methodology. **Kazushige Kawai:** data curation, investigation, writing – review and editing. **Yusuke Suwa:** investigation, writing – review and editing, data curation. **Kazuki Ueda:** data curation, investigation, writing – review and editing. **Shigeru Yamagishi:** data curation, investigation, writing – review and editing. **Takeshi Naitoh:** data curation, investigation, writing – review and editing, supervision. **Tatsuki Noguchi:** data curation, investigation, writing – review and editing.

## Funding

The authors have nothing to report.

## Ethics Statement

The protocol for this study was approved by the institutional review boards of the participating hospitals. Study regarding long‐term prognosis of the cases recruited in “Prospective Study Investigating Laparoscopic Surgery for Locally Advanced Rectal Cancer with Evaluation of CRM and TME quality: PRODUCT trial” was registered in the University Hospital Medical Information Network (UMIN http://www.umin.ac.jp/ctr/index.htm).

## Consent

Written informed consent was obtained from all patients.

## Conflicts of Interest

A. Hamabe receives honoraria for lectures from Merck BioPharma Co. Ltd., Johnson & Johnson, Medtronic, and Fujifilm. M. Ito has financial relationships with the following entities outside the submitted work: Asahi Intecc Co. Ltd., Jmees Inc., Surg storage Co. Ltd., Indivumed GmbH, CareNet Inc., Livsmed Inc., Guardant Health Inc., Johnson & Johnson K.K., Applied Medical Japan, Covidien Japan, Takano Co. Ltd., Nition Co. Ltd., EBM Co., Muranaka Medical Instruments Co. Ltd., BMG Inc. and Kotobuki Medical Inc. M. Inomata has financial relationships as honoraria from Covidien Japan, Medicaroid Corporation, Johnson & Johnson, and research funding for collaborative studies with Olympus Corporation and SB Kawasumi Laboratories Inc. Y. Hirano has financial relationships as honoraria from Intuitive Surgical and Asensus Surgical. T. Naitoh has financial relationships with following entity outside the submitted work: honoraria from Johnson & Johnson, Covidien Japan, Stryker, Olympus, Takeda Pharmaceutical, Taiho Pharmaceutical, Chugai Pharmaceutical, and Eli Lilly. T. Naitoh also receives research grants from Terumo, and TOPPAN holdings.

## Data Availability

The data that support the findings of this study are available on request from the corresponding author. The data are not publicly available due to privacy or ethical restrictions.
